# Effective access to health care in Mexico

**DOI:** 10.1186/s12913-022-08417-0

**Published:** 2022-08-12

**Authors:** Rocio Garcia-Diaz

**Affiliations:** Tecnologico de Monterrey, Ave. Eugenio Garza Sada 2501, Monterrey, N.L. Mexico

**Keywords:** Out-of-pocket health expenditures, Access, Medicines, Health systems, Econometric methods, I21, I22, D24, R12, I21, I22, D24, R12

## Abstract

**Objectives:**

This paper assesses the impact of effective access on out-of-pocket health payments and catastrophic health expenditure. Effective access cannot be attained unless both health services and financial risk protection are accessible, affordable, and acceptable. Therefore, it represents a key determinant in the transition from fragmented health systems to universal coverage that many low- and middle-income countries face.

**Methods:**

We use a definition of effective access as the utilization of health insurance when available. We conducted a cross-sectional analysis using the 2018 Mexican National Health Survey (ENSANUT) at the household level. The analysis is performed in two stages. The first stage is a multinomial analysis that captures the factor associated with choosing effective access against the alternative of paying privately. The second stage consists of an impact analysis regarding the decision of not choosing effective access in terms of out-of-pocket (OOP) health payments and catastrophic health expenditures (CHE). The analysis corrects for both the decision to buy insurance and the decision to pay for health care.

**Results:**

We found that, on average, not choosing effective access increases OOP health payments by around 2300 pesos annually. Medicine payments are the most common factor in this increase. Nevertheless, outpatient and medicines health care are the main drivers of the increase in OOP health payments in all insurance beneficiaries. Not having effective access increases the probability of CHE health expenditures by 2.7 p.p. for the case of Social Security Insurance and 4.0 p.p. for Social Government insurance. Household enrolled in *Prospera* program for the poor are more likely to choose effective access while having household heads with more education and assets value does the opposite. Diabetes illnesses are associated with a higher probability of effective access.

**Conclusion:**

Improving effective access is a middle step that cannot be disregarded when seeking universal coverage because OOP health payments and catastrophic outcomes are direct consequences. Public insurance in general, has around 50% effective access which remains a challenge in terms of health services utilization and health public policy design, calling for the need of better coordination across insurance types and pooling mechanisms to increase sustainability of needed health services.

**Supplementary Information:**

The online version contains supplementary material available at 10.1186/s12913-022-08417-0.

## Background

Universal health coverage is one of the main policy objectives in developing economies. Universal health coverage means that all households have access to health care, when they need them without incurring in financial hardship [1]. To tackle this objective, many developing economies have evolved into a segmented and fragmented health system where many insurance types co-exist [2]. Fragmented because many non-integrated subsystems co-exist, such as contributory social security, non-contributory government social security and the private sector and all insured and delivered health services [3]. On the other hand, segmentation means that these subsystems follow different sources for financing, rules for affiliation and provision intended for different segments of the population [4].

This fragmentation and segmentation of coverage is paired with an uncoordinated private sector that responds to the lack of satisfaction and capacity in the public sector, making the task of fair and increasing private financing across populations difficult and contributing to the social inequalities in the region [5]. Latin America and the Caribbean have the highest rate of people with out-of-pocket expenditures exceeding 10% or 25% of total household consumption or income [1].

To capture whether households with social insurance needing health services obtain them and how much they pay for them, we use the concept of effective access in the utilization of health insurance as proposed by [6]. For that, we combine health insurance affiliation with health care utilization among households. In this sense, we say that individuals with public insurance who consistently make use of private services through direct payments do not have effective access. This paper then estimates the impact of effective access on catastrophic health expenditures (CHE) and on out-of-pocket (OOP) health expenditures in Mexican households.

The analysis is performed in two stages. In the first stage, we use a multinomial analysis to describe the demographic characteristics and identify factors associated with choosing effective access against the alternative of paying privately. Our model accounts for the endogeneity of a household’s decision to enroll in a social insurance program [7–10]. The second stage is an impact analysis of these households’ decisions on OOP health expenditure and CHE. It uses selection-correction methods for the decisions to buy health care and to buy insurance [11, 12]. To enable the operationalization of effective access to health care, we use the 2018 ENSANUT, at the household level. We employ the concept of OOP health payments during a year and its components: Medicines and outpatient and inpatient health care.

Health financing remains an empirical question that justifies a systematic analysis to provide evidence of the practical effects of social insurance. In this regard, [12] analyzed the effect of health insurance on medicines and found marked heterogeneous effects across insurance types. There is a series of studies that find positive results for social insurance, in terms of either OOP health expenditures or catastrophic health expenditures when compared to the uninsured subpopulation [7, 8, 13, 14]. Nevertheless, despite finding some encouraging results, there is evidence that not all persons who have insurance end up not using it, that benefit packages are sometimes limited, and households may experience financial hardship when paying for health care [15, 16].

We use the concept of utilization of health care as realized access [17], that is, when people take the steps that enable them to make contact and obtain health care. Effective access to social protection is determined by factors such as the availability, price, and quality of health resources a patient may experience [18]. It represents crucial transitions and where barriers can be revealed. This paper adds to this idea and to some studies in Latin America which find that insurance coverage alone does not provide a guarantee of a reduction in OOP health expenditures [12, 16], especially when households face, at first sight, cheap and convenient private alternatives [15]. It has been found that that two-thirds of the users of doctors’ offices adjacent to private pharmacies have medical insurance. We aim to shed some light on why some households with insurance choose to pay privately and the consequences of those decisions over time in terms of OOP health expenditure and catastrophic health expenditure.

### Health insurance in Mexico

Mexican health systems, according to employment characteristics, can be divided into the formal sector and the informal sector [19]. Formal sector employees are enrolled by law in the Mexican National Social Security (IMSS, for its acronym in Spanish), at the federal level. This social security is financed by employer and employee payroll taxes by legal mandate and by the government. In this social security institution, the tripartite scheme is usually maintained unless the employer is the government itself. These are other parallel government-run institutions for those who work in the federal or state government, the state oil company (PEMEX, for its acronym in Spanish) and the military (SEDENA, for its acronym in Spanish). These institutions provide a series of health benefits for their members, including a broad package of pre-paid interventions, treatment by general practitioners, and all the medicines for the specified treatments.

Informal sector workers receive basic health services through the Ministry of Health, which oversees sanitation responsibilities and basic health for all the population. There are government-sponsored (GS) health insurance schemes for informal workers and uninsured population [20]. The most important program for the period of this analysis was the Social Health Protection System, or *Seguro Popular*, was a voluntary government-sponsored insurance focused on helping the poor who do not have access to any social security. *Seguro Popular* was financed primarily by federal and state governments. Although the program was intended to be a pre-payment health insurance where beneficiaries’ contributions to the annual payments were based on their ability to pay, it never happened [3, 21]. GS health insurance schemes also include other programs for the poor and non-insured, such as IMSS-Prospera and, recently, the Institute for Health for Wellbeing (INSABI), which has replaced Seguro Popular and aims to establish a fully funded and integrated public health network.

The reports on ENSANUT, indicate that for 2018, at the individual level, 37% of the population reported having GS health insurance, 42.4% reported having SS health insurance, and 18.7% reported being insured [22]. At the household level, the story is different. Despite efforts to offer financial protection for all populations, in 2018 14.6% of households in the population still report having no insurance [23]. In addition, 25.8% of the households stated that at least one member in the household had no insurance or had multiple insurance types [24].

It is also important to note that the package of services and coverage across illnesses is very different across insurance types. Social insurance institutions perform this function through their own pharmacies at no cost. However, access to medication and health services infrastructure is still incomplete. In terms of medication, it has been reported that 86% of IMSS users who received prescriptions for medication in any medical unit obtained all the prescriptions in the same place, compared to 63% for *Seguro Popular* users. On the other hand, the supply of medications within the pharmacies with an adjacent physician has been found to be 74% [12, 23]. The high specialty health infrastructure, such as tertiary care hospitals, varies widely across insurance types. Patients often need to travel long distances or wait long periods of time to get the service they need within their own institution due to a very segmented market. A recent study found that 50% of adult Mexicans living in urban areas, live close to a hospital that does not belong to their insurance scheme.

## Methods

### Data

Data for this study came from the 2018 Mexican National Health and Nutritional Survey (ENSANUT-2018). The ENSANUT is a probabilistic survey that employed a multistage stratified cluster sampling design. The survey is representative at the national, regional, and state levels, including urban and rural strata. The sampling frame was taken from the 2010 Population Census primary sampling units. ENSANUT-2018 data were collected in 42, 699 households, between July 2018 and June 2019, with a response rate of 85.50%. These households represent an estimated 33, 368, 826 households across all 31 Mexican states and Mexico City, out of which 39,032 households were included in the sample (households with incomplete information on income, expenditure, and illness variables were excluded from the sample).

#### Exposure variables

Households are identified according to four types of household members’ medical insurance: (1) Social Security (SS), (2) Government Social Insurance (GS), (3) Mixed affiliation, in this case, household members have different insurance types, including private, or some members are uninsured, (4) households with no financial protection. For the case of SS and SG insurance, we used a strict definition of household affiliation selecting only those households in which all members are covered. On doing so, we first identify individuals with either SS or SG social insurance. Then we aggregate all members in the household with their corresponding insurance category and obtain a ratio using the household size. The households selected for the corresponding insurance category are those with the ratio equal to 1.

The different access categories in Table 1 are defined by the combination of two indicators: insurance affiliation and health service utilization. The insurance affiliation follows the question, do you have access to health service in …? Health service utilization follows the question, when you have health problems, where do you regularly go for health services? Once we have identified these two variable indicators, we identify households that are beneficiaries of different health insurance affiliations in two categories: Those which regularly use their health services provided at their affiliation institution and those that opt for not using their health services and regularly choose to pay privately.

**Table 1:**
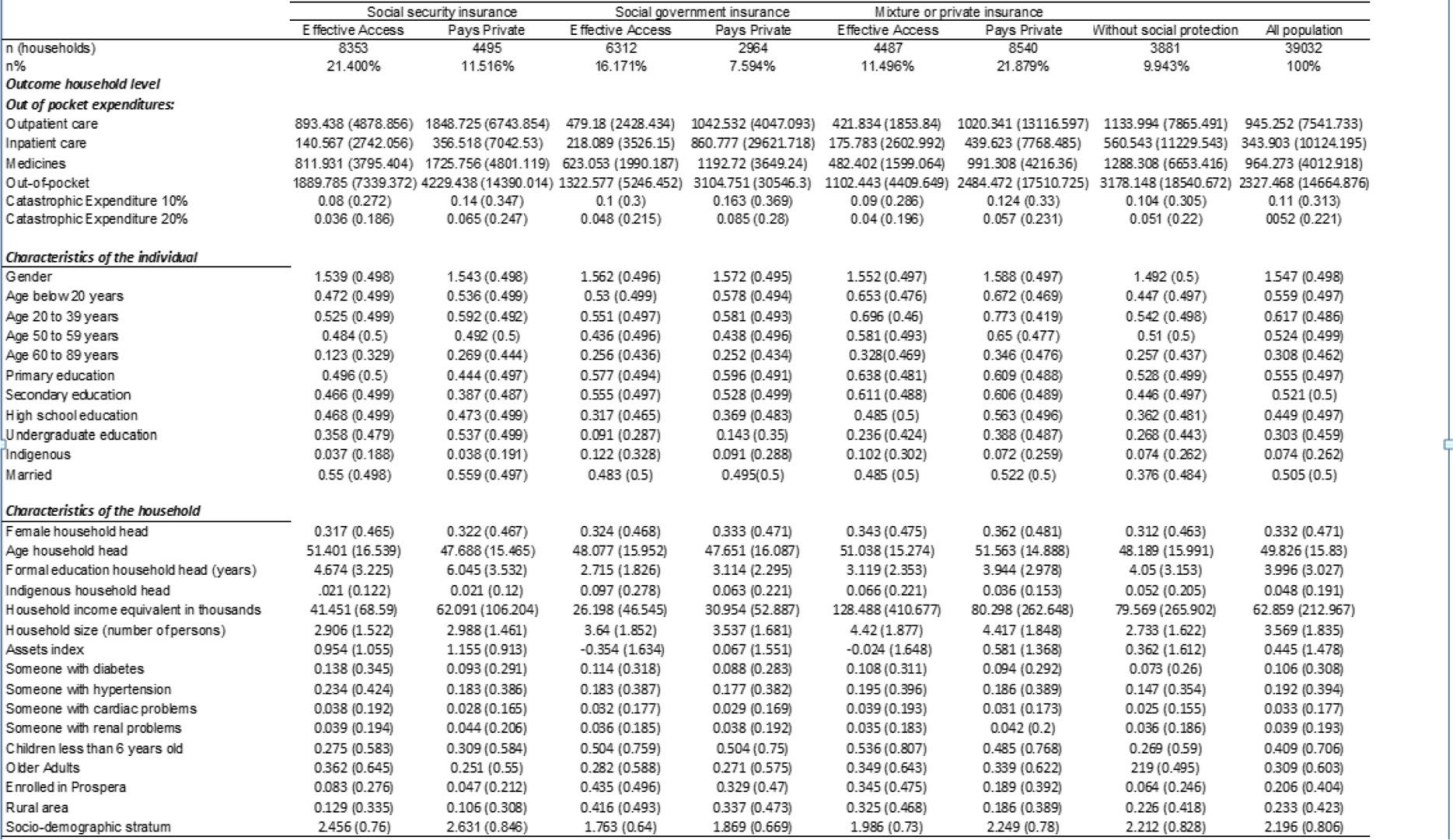
Main characteristics of the sample

Source: Author’s calculations based on ENSANUT 2018

Standard error in parenthesis. The asset index is constructed using a principal component analysis using the identification of certain items in the household, household size, number of older adults and children under 6 years of age, and the identification of major illnesses

Out of the 39,032 households in our sample, 33% reported Social Security (SS) insurance, and 35% reported non-effective access by choosing to pay privately for health care. Government-sponsored (GS) Insurance was reported by 23.76% of the sample and, within the same, 32% reported consistently using private services. Households with multiple insurance types or some uninsured members represent 33.37% of the sample, out of which, 65% have reported paying privately for health services. Finally, 9.94% of the sample reported having no health insurance (Table 1). Generally, we can identify small socioeconomic and development differences according to different household insurance types and the choice whether to have effective access. The medicine component with respect to total OOP health payments is higher in those households that having health insurance choose to pay privately for health care. Similarly, those that have non-effective use of their health services report larger proportions in outpatient health care in all insurance categories. Both components, medicine and outpatient health care, are the main drivers of total OOP health expenditure. Education also reports a variation for those households preferring effective access. For the beneficiaries of SS health insurance, the more educated the household head, the more likely they are to pay privately for health care. That situation is different for those having GS health insurance, in which at any education level they will opt for private alternatives. Both being indigenous or married would produce no meaningful variation between having an effective access or not. Higher-income households will be more likely to opt for private health care, for both GS and SS health insurance. In the case of mixed insurance, that difference is reversed. Having members with health problems (either diabetes, hypertension, renal or cardiac) is associated with more effective use of health services provided by their institutions. Generally, enrolling in *Oportunidades* and living in a rural area predispose households to pay privately for health care, where geography and distance contribute to their health-care decisions.

#### Outcome variables

The outcome variables are the probability of paying out-of-pocket (OOP) health payments. OOP health payments are the amount of money paid by individuals to purchase health services and medicines when individuals have a health-care need. We construct total out-of-pocket health expenditure according to the sum of its three main components. Medicines account for 40% of the total private expenditure for all the population. Outpatient expenditures account for diagnostic tests, doctors’ fees, and other medical services, which, on average, represent 41% of the total private health expenditures. Inpatient expenditures are those related to hospitalizations payments, which account for 19% of the total private expenditure in the population. This corresponds to the total health expenditure incurred by all household members during the last three months of 2018. Then, it is multiplied by four to obtain the annual OOP health expenditure, since the survey reports this type of expenditure in a quarterly form. This is consistent with the official SDG indicator as total income or consumption and ability-to-pay measure [25]. There are other studies that have suggested, for poorer economies, focusing on essential items of spending, such as food, and overlooking other non-discretionary spending related to more durable goods. Here, we have chosen total income as a proxy of our ability to pay given that, in Mexico, expenditure categories other than food are relatively more relevant.

The probability of the incidence of catastrophic health expenditure (CHE) in households is another outcome variable in the analysis. To calculate the incidence of catastrophic health expenditure we use the budget share method, which calculates the ratio of OOP health expenditure in the household to its total income or expenditure [26]. The threshold we use to identify catastrophic health expenditure is 20%, as previously used for the sustainable development goals (SDGs), monitoring catastrophic spending using the budget share method [27]. Following the definition of CHE, it will take the value of 1 if the household’s health expenditure related to total income exceeds 20% of total household income and 0 otherwise [26].

It has been recognized that total consumption is preferred as the denominator because households would be expected to have other resources available to pay compared to total income [28]. However, ENSANUT- 2018 no longer provides all expenditure categories, except for the health-related categories, and we proxy total income with the sum of labor income for all members of the household. This, in consequence, may cause two effects. On the one hand, it will overestimate our catastrophic health expenditure indicator in some households in the analysis because not all income sources are included. On the other, it may underestimate our catastrophic health expenditure indicators by not including households working in the informal sector of the economy. It also considerably reduced our sample size in Table 4 because many households did not report income information. The catastrophic health expenditure indicator at 10% total household income is on average 10%, which is higher than those reported in the global monitoring report on financial protection in health 2019 of 1.6–2% for 2016 [1], Annex 14.

#### Econometric approach

The data analysis is performed in two stages. In the first stage, we use the sample to describe sociodemographic characteristics associated with households choosing effective access or not. The second stage involves an impact analysis of effective access on OOP health payments and catastrophic health expenditures. All models are adjusted for household head characteristics, and the socioeconomic and demographic characteristics of the households, as well as geographical and local characteristics.

**Fig. 1:**
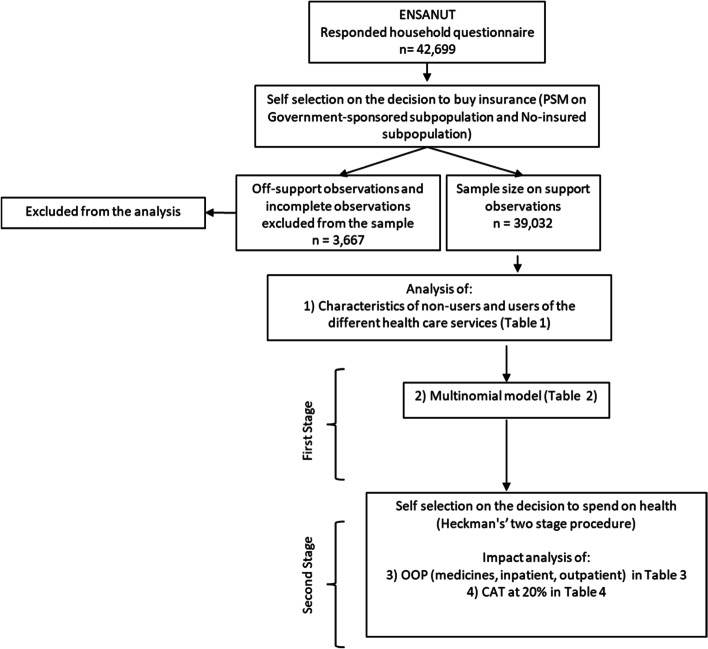
Sample selection in the analysis

There are two potential biases in the analysis. First, there is a decision to spend on health care, since certain household characteristics may increase the probability of spending privately [8, 11]. This is a potential bias for all households seeking health care in the analysis. The second potential bias is related to the decision to buy insurance or not. That is, individuals with certain characteristics or those that expect to require more medicines or medical treatments in the short run will be more prone to buy insurance. This potential bias is corrected only for the case of *Seguro Popular*, where individuals may enroll voluntarily, in contrast to mandatory enrollment of SS insurance [12, 29]. Therefore, SS, mixed-affiliation, and without insurance are separated for those *Seguro Popular* enrolled households. Figure 1 explains the correction biases for the sample and the two-stage econometric planning for the analysis.

In the first stage of our analysis, we control for the individuals’ decision to buy *Seguro Popular* insurance. We apply the propensity score matching method [9], which has been found useful for minimizing the selection bias associated with non-experimental data. The objective of matching is to find a large group of non-participants. Individuals that are like the participants in observed pre-treatment characteristics, $X$, are called individuals on-support. Assuming the two groups do not differ in observable characteristics, we can say that differences in outcomes in both groups can be attributed to the treatment program. The use of balancing scores, $b\left(X\right)$, function of the relevant observed co-variates $X$ that would be expected to be equal for both groups if the conditional distribution of $X$ is independent of treatment assignment [30]. The propensity score matching is the probability of participating in the program given observed characteristics $X$. The $X$ variables selected to estimate the *Seguro Popular* insurance program score were those previously used by [31] in calculating its point scores to determine individuals’ enrollment in the *Seguro Popular*. The variables include individual characteristics, such as gender, age, education, being indigenous, and household characteristics, such as, assets index,^1^ household size and geographical variables (see Appendix, Table A.1).

To verify the balancing, we tested whether the average score between control and comparison units within each stratum were statistically the same. Once the strata were balanced, we perform individual mean t-test between controls and comparisons for each of the variables used to predict the score (see Appendix, Table A.2). Then we chose those households that belong to the common support region, where the balancing score has positive density for both treatment and comparison units, that is, where there was an overlap between the treatment and comparison groups). In this way, we ended up with a sample size of 134,167 individuals that constitute 39,032 households, from the initial sample of 158,838 individuals that represented 42,699 households, having discarded the households that do not have common support (See Appendix, Figure A.1).

The sample after selection bias correction to buy insurance served to describe factors associated with the probability of choosing an effective access. We propose a multinomial logistic model to find the probability of a household choosing a health care provider $j$ in comparison to certain option $k$, that is,

$$\frac{{P}_{ij}}{{P}_{ik}}=\frac{exp\left({\alpha }_{j}+{\beta }_{i}{X}_{ij}\right)}{exp\left({\alpha }_{k}+{\beta }_{i}{X}_{ik}\right)}$$

Because our intention is focused on paying privately as an alternative, this option was chosen as our comparison base. The health providers are SS insurance, SG insurance and mixed insurance. We omit households without insurance because they have no other alternative than to pay privately. The analysis includes independent variables suggested in the literature for the use of health services [15, 32]. The variables include characteristics of the household head, such as being female, age, and marital status. Education of the household head is a categorical variable (no education, primary education, secondary education, high school, and college education). Other situational characteristics of the household, such as asset index, are constructed using a principal component analysis. The variables employed are household size, number of older adults, children under six years of age, and the identification of major illnesses such as diabetes, hypertension, cardiac, and renal problems in the members of the household. These variables are presented in Table 1.

In the second stage of the analysis, we address the selection bias of spending on health care. Following [11, 12], we implement the Heckman twostep expenditure procedure that relies on normality assumption and is considered robust. Formally, let ${y}_{1}^{\prime }$ be the latent variable denoting (outcome), be either, the level of OOP health expenditures, or the level of catastrophic health expenditures at 20%. These outcomes are observed if households decide to spend on health care. This decision can be expressed as another latent variable, ${y}_{1}^{\prime }$, that assumes a value of ${y}_{1}^{\prime }=1$ if the household spends on health care and ${y}_{1}^{\prime }=0$, otherwise. Let ${Z}_{i}$ denote the vector of exogenous and fully observed regressors which can be used in both equations to examine its associations with outcome and selection variables. The conditional expectation for the two-step method can be written as:

$$E\left({y}_{2}|{{z}, \, y}_{1}^{\prime }>0\right)={{z}}_{2}{\beta }_{2}+{\sigma }_{12}\lambda \left({x}_{1}{\beta }_{1}\right)$$

where ${\sigma }_{12}$ is error covariance and $\lambda \left({x}_{1}^{^{\prime}}{\beta }_{1}\right)$ the inverse of the Mill’s ratio or the non-selection estimates obtained by a probit regression of ${y}_{1}$ on ${x}_{1}^{^{\prime}}$. The standard errors for the regression coefficients in the outcome equation are computed allowing for the estimation error of $\lambda \left({x}_{1}^{^{\prime}}{\beta }_{1}\right)$. The selection equation includes per capita public expenditure at the state level. The outcome equation includes an appropriate covariate vector we search in the literature (cite). It includes household head characteristics, such as, age, gender, education, and being indigenous. Household socioeconomic variables, such as household size, presence of older adults and presence of children less than six years old, are also included. Health problems among household member are also considered, such as, diabetes, cardiac problems, hypertension, and renal problems. It also contains geographical variables that identify if a household is in a rural area, and the deprivation level of the area, and, finally, if a household is enrolled in the social program *Oportunidades*, which is the main conditional cash transfer program in Mexico for alleviating poverty.

## Results

Table 2 shows the decisions by households to choose an effective access or paying privately, modelled by a multinomial logistic regression. The outcome variable is choosing an effective access or paying privately, which is modelled as a linear combination of the predictor variables. The column denoted “SS vs. Private” refers to a household’s preference for choosing an effective access at an SS insurance facility in comparison to paying privately, which serves as a reference category. The second column “SG vs. Private” corresponds to household preference for choosing government-sponsored insurance in comparison to paying privately, and the last column to households that prefer private or mixed insurance.

**Table 2:**
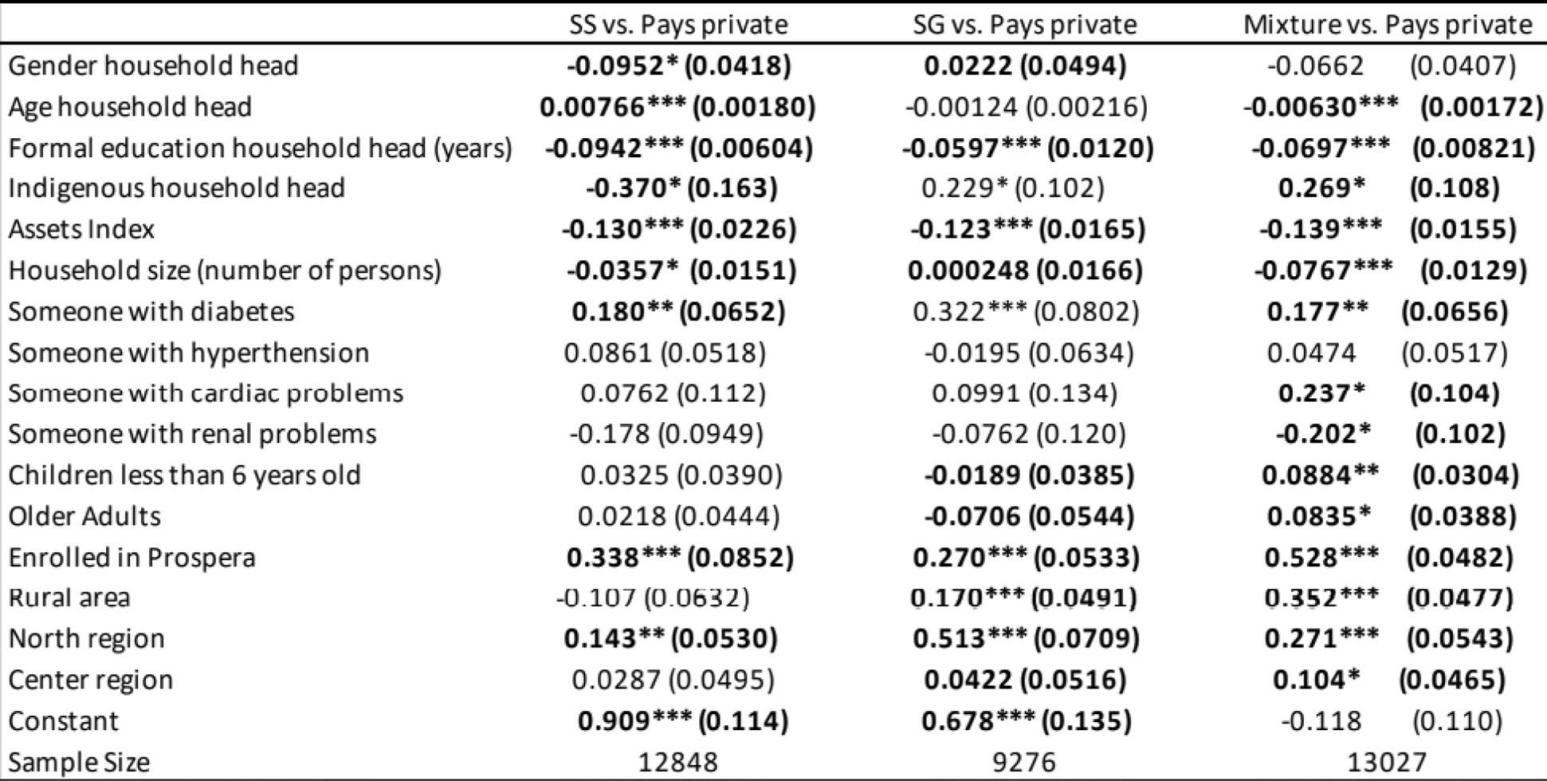
Multinomial probit model

Source: Author’s calculations based on ENSANUT 2018

*Significance at 10%, ** significance at 5%, *** significance at 1%

Bold values correspond to estimatesthat are significant at the 10%, 5% or 1% level

We can observe that, among the most common health problems in the population, households whose members have diabetes issues seek health care in their corresponding health insurance facilities, which is the case for all categories. On the other hand, for the population with multiple insurance schemes, having cardiac issues was associated with an effective access, while members with renal problems are more likely to pay privately. This is probably related to the fact that coverage of certain illnesses is a major differentiating factor across insurance types. Regarding sociodemographic characteristics, female household heads prefer effective access, in the case of SG insurance, but they are more likely to pay privately in the case of SS. The older the household head, the more likely they are to utilize their service institution for the case of SS health insurance, while the case of having no full coverage in the house increases the probability of paying privately. In this subpopulation, as people age and more health issues emerge, households tend to pay more privately since coverage is not complete. Household head education is positively associated with not having an effective access and opting for private alternatives across all insurance categories. With respect to household composition, having more household members increases the probability of paying privately, except for the case of SG insurance. In the case of mixed health insurance, as the number of members increases the chances of having uninsured members also increases. Having children younger than six years old or older adults decreases the probability of taking effective access and opting for paying privately for the case SG insurance, while the opposite happens for mixed insurance. Chronic illnesses are a result of the aging population and tend to be more expensive and last for longer periods; therefore, households tend to circumvent increasing their health budget by attending their insurance facilities to obtain the services they need. Geographically, households living in rural and poor areas were associated with choosing effective access in the case of the SG and mixed insurance categories. SS was not statistically significant; this could be related to issues of location and distance in their utilization of SS health infrastructure, which is mostly available in urban localities.

Table 3 presents the selectivity correction models for non-effective access in total out-of-pocket health expenditure. The unweighted effects are presented in the upper panel and weighted effects in the lower panel. Column one corresponds to households having SS insurance, the second column to those that have government insurance, and the last one to households with multiple or private health insurance. The outcomes of interest are total OOP health expenditure and its components: Medicines and inpatient and outpatient health expenditures.

**Table 3:**
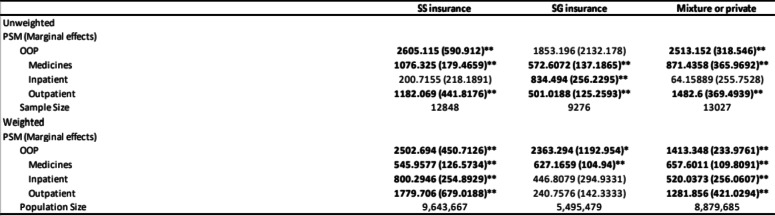
PSM models for paying private on OOP health expenditure

Source: Author’s calculations based on ENSANUT 2018

*Significance at 10%, ** significance at 5%

a. CAT defined asOOP spending greater than 20% oftotal equivalent household income

b. Annualized OOP health expendituremeasured in Mexican pesos of 2018

Bold values correspond to estimatesthat are significant at the 10%, 5% or 1% level

For the weighted analysis, we can observe that choosing to pay privately when having SS or mixed health insurance increases the total OOP health expenditure by 2,605 in the case of SG insurance and 2,513 (in pesos, per year) for the case of multiple or private health insurance. Choosing regularly to pay privately for medicines increases health expenditures by 657 pesos per year in the case of Mix insurance, 627 pesos for the case of SG insurance, and 545 pesos for the case of households with different insurance or private insurance. Outpatient health expenditure is statistically significant in all insurance categories. Households, pay on average pay 1,182 pesos per year more for the case of SS insurance and 502 pesos for the case of SG insurance and 1,482 for mixed insurance. The tendencies remain mostly the same for the unweighted effects.

Table 4 presents the marginal effects of not having effective access to catastrophic health payments, measured as households that spend more than 20% of their income on OOP health payments while controlling for all the households’ characteristics listed in Table 1. The unweighted effects are presented in the upper panel and weighted effects in the lower panel. The first column corresponds to those households having SS insurance, the second column corresponds to SG insurance, and the third column corresponds to those having mixed health insurance. The change in the number of observations with respect to Table 3 sample sizes are due to missing values for the equivalent household income variable. We can see a variation in the probability of CHE among insurance types. For households that choose to pay privately there is an increased probability in CHE of 2.0 p.p. for the case of SS insurance, and 4.0 p.p, for the case of SG insurance. When population weights are added, the direction and values of the coefficients remain approximately the same, 2.7 p.p. for the case of SS insurance, but the coefficient for SG loses significance.

**Table 4:**
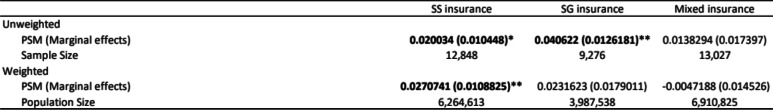
PSM models for paying private on CAT health expenditure

Source: Author’s calculations based on ENSANUT 2018

*Significance at 10%, ** significance at 5%

a. CAT defined as OOP spending greater than 20% of total equivalent household income

b. Annualized OOP health expenditure measured in Mexican pesos of 2018

Bold values correspond to estimates that are significant at the 10%, 5% or 1%

## Discussion and conclusion

This study is relevant in the context of fractioned health systems common in developing economies where different health insurance types co-exist. It points out that, that opting out of the health service alternatives provided by their health insurance constitutes a common response among families in Mexico (40.97% in our sample does). Non-effective health access represents a higher proportion of OOP health expenditure and a higher risk of CHE, which impacts households’ economic situations.

Government-sponsored insurance has been widely employed to achieve universal health coverages across developing countries [33, 34]. In Mexico, those efforts have not provided coverage for the entire population, even though it was announced in 2012 [29]. Government efforts not only were unable to reach the number of affiliations needed, but also the specified packages of services and medicines denied households the medical care they needed [35]. Our findings echo those of other studies in that just having social insurance alone may not have a huge impact on OOP health expenditures, nor provide a guarantee of OOP health reduction across households [36, 37]. This result has been previously supported by other research in Mexico and other countries [32, 38, 39]. Here, we find that is not just about membership in a financial protection scheme, but also choosing to make it effective or not. Moreover, when all household members are fully insured, beneficiaries are found to be more likely to pay privately.

There may be many reasons why households choose to pay privately. The specific sociodemographic characteristics for opting out of their health service alternatives are related to age, and being positively associated when younger children are present, and negatively for the presence of older adults. We find evidence that some illnesses, such as renal illnesses, are more likely to induce private payments in the case of mixed insurance and not in other insurance types. Households with members who have diabetes tend to pay long-term and expensive treatments and are more likely to use their insurance services, across all insurance categories. Yet, there is no evidence of this is happening in other insurance types, which prompts attention on differences in medicines and coverage across illnesses in insurance alternatives. Therefore, there is a need to address changing populations and epidemiological profiles, with more attention on treatment conditional on illness, as also pointed out by [40]

In our analysis, medicines constitute the most common driver of private health financing. Households that have insurance and opt for paying privately spend on average between 1400 to 2,600 pesos more than those that use their insurance alternatives. The shortage of medicines and no-coverage of certain illnesses are a contributing factor to private spending. Insurance users, who attend doctor appointments and get diagnosis services, find shortages of medicines, and need to buy them privately. The new sponsored-government insurance INSABI efforts on centralizing purchases of medicines at federal level in order to reduce state discretion on spending could reduce the effect of households paying-privately if all insurance follow coordinated protocols for illnesses associated with the population epidemiological profile that does not vary much across insurances. Otherwise, this public health sector’s struggle to fulfill an increasing demand for health services and medicines is going to be met by an un-coordinated private service alternatives such as the health services of doctors adjacent to private pharmacies (DAPPS). There is evidence that DAPPS users are being prescribed an excessive number of medicines compared to the standard in health services. This reflects our findings that paying privately is related to an increase in medicine costs and services, especially ambulatory services across all insurance types.

An important consideration of this analysis is that the proportion of uninsured households or partially uninsured households, where at least one member is without insurance, is still very high (43.3%). This percentage is similar to the 40% of total households that were partially insured and uninsured in 2012 [24]. It indicates the need for policies to ensure health care for the older population, especially in those households with partial insurance. In the same way, older adults are prone to longer illnesses and more expensive health treatments and are forced to use private alternatives. INSABI’s focus on providing services no-coverage for everyone will be ideal but need to avoid double counting of individuals with multiple affiliations for a better use of public resources through better fundraising and pooling mechanisms. Otherwise, these changes can re-inforce inequalities in health care as [41] suggests.

There are some important limitations in this study. We have no information on diagnoses, but just four major illnesses in the Mexican population. The information on the expenditure survey is not previously related to a particular illness or previous diagnosis. The out-of-pocket information in the survey has a recall period of three months, which conveys a recall issue associated with sub-estimation on the level of OOP expenditures and CHE. The information on health expenditure is at the household level and even though there is information at an individual level with a recall period of 15 days, this information is related to a health-need event. Health need is mostly associated with curative health care, and not preventive and palliative health care that are also important in total OOP health expenditure, which constitutes the basis of this analysis.

## Supplementary Information

**Additional file 1.**

**Additional file 2.**

**Additional file 3.**

## Data Availability

Data and materials are available by emailing Dr. Rocio Garcia-Diaz at rociogarcia@tec.mx. Data was obtained from the following source: the 2018 Mexican National Health Survey (ENSANUT) is publicly available at https://www.inegi.org.mx/programas/ensanut/2018/default.html#Microdatos, following the link *Descargar todos los archivos* (Download all files). There are no administrative permissions required.

## Abbreviations

OOP: Out-of-pocket
ENSANUT: Mexican National Health Survey for its acronym in Spanish
CHE: Catastrophic health expenditures
IMSS: Mexican National Social Security, for its acronym in Spanish
PEMEX: Mexican oil company, for its acronym in Spanish
SEDENA: Mexican military, for its acronym in Spanish
SS: Social Security
GS: Government-sponsored
DAPPS: Doctors adjacent to private pharmacies

## Footnotes

1 An assets index is estimated using principal component analysis over a range of indicators that indicate the economic well-being of a household. The indicators we use are: type of flooring, the presence of a gas cooker, availability of piped water, the type of toilet, heater and/or air conditioning. It has been recognized as a good proxy for income or expenditure and is more stable over time [42].

## References

[1] WH Organization (2019). Primary Health Care on the Road to Universal Health Coverage 2019 Global Monitoring Report Executive Summary.

[2] Knaul F, Frenk J (2017). Health Insurance In Mexico: Achieving Universal Coverage Through Structural Reform. Health Aff.

[3] del Valle A (2021). The effects of public health insurance in labor markets with informal jobs: Evidence from Mexico. J Health Econ.

[4] Bossert T, Blanchet N, Sheetz S, Pinto D, Cali J, Cuevas RP. Comparative Review of Health System Integration in Selected Countries in Latin America. IDB Technical notes. Inter-American Development Bank. 2014.

[5] Siqueira M, Coube M, Millett C, Rocha R, Hone T (2021). The impacts of health systems financing fragmentation in low- and middle-income countries: a systematic review protocol. Syst Rev.

[6] Gutiérrez J, García-Saisó S, Dolci G, Ávila M (2014). Effective access to health care in Mexico. BMC Health Serv Res.

[7] Sosa-Rubí SG, Galárraga O, López-Ridaura R (2009). Diabetes treatment and control: the effect of public health insurance for the poor in Mexico. B World Health Organ.

[8] Ávila-Burgos L, Serván-Mori E, Wirtz VJ, Sosa-Rubí SG, Salinas-Rodríguez A (2013). Efectos del Seguro Popular sobre el gasto en salud en hogares mexicanos a diez años de su implementación. Salud Pública Méx.

[9] Trujillo AJ, Portillo JE, Vernon JA (2005). The Impact of Subsidized Health Insurance for the Poor: Evaluating the Colombian Experience Using Propensity Score Matching. Int J Heal Care Finance Econ.

[10] Bleich SN, Cutler DM, Adams AS, Lozano R, Murray CJL (2007). Impact of insurance and supply of health professionals on coverage of treatment for hypertension in Mexico: population based study. BMJ.

[11] Galárraga O, Sosa-Rubí SG, Salinas-Rodríguez A, Sesma-Vázquez S (2010). Health insurance for the poor: impact on catastrophic and out-of-pocket health expenditures in Mexico. Eur J Health Econ.

[12] Wirtz VJ, Santa-Ana-Tellez Y, Servan-Mori E, Avila-Burgos L (2012). Heterogeneous Effects of Health Insurance on Out-of-Pocket Expenditure on Medicines in Mexico. Value Health.

[13] Gakidou E, Lozano R, González-Pier E, Abbott-Klafter J, Barofsky JT, Bryson-Cahn C (2006). Assessing the effect of the 2001–06 Mexican health reform: an interim report card. The Lancet.

[14] Garcia-Diaz R, Sosa-Rubi SG, Sosa-Rub SG (2011). Analysis of the distributional impact of out-of-pocket health payments: Evidence from a public health insurance program for the poor in Mexico. J Health Econ.

[15] Pérez-Cuevas R, Doubova SV, Wirtz VJ, Servan-Mori E, Dreser A, Hernández-Ávila M (2014). Effects of the expansion of doctors’ offices adjacent to private pharmacies in Mexico: secondary data analysis of a national survey. BMJ Open.

[16] Wagstaff A, Dmytraczenko T, Almeida G, Buisman L, Eozenou PH-V, Bredenkamp C (2017). Assessing Latin America’s Progress Toward Achieving Universal Health Coverage. Health Affair.

[17] Frenk J. Concept and measurement of accessibility. Salud Pública de México. 1985;27(5):438-53. 4081889

[18] Haddad S, Fournier P (1995). Quality, cost and utilization of health services in developing countries. A longitudinal study in Zaïre. Soc Sci Med.

[19] Dantés OG, Sesna S, Becerril VM, Knaul F, Arreola M, Frenk J (2011). Sistema de salud de México. Salud Pública Méx.

[20] Brown CJ, Pagán JA, Rodríguez-Oreggia E (2005). The decision-making process of health care utilization in Mexico. Health Policy.

[21] Lakin JM (2010). The End of Insurance? Mexico’s Seguro Popular, 2001–2007. J Health Polit Policy Law.

[22] Shamah-Levy T, Vielma-Orozco E, Heredia-Hernández O, Romero-Martínez M, Mojica-Cuevas J, Cuevas-Nasu L, Santaella-Castell JA, Rivera-Dommarco J. Encuesta Nacional de Salud y Nutrición 2018-19: Resultados Nacionales. Cuernavaca, México: Instituto Nacional de Salud Pública; 2020.

[23] Block MÁG, Morales HR, Hurtado LC, Balandrán A, Méndez E; World Health Organization. Mexico: Health system review; 2020.

[24] Urquieta-Salomón JE, Villarreal HJ (2016). Evolution of health coverage in Mexico: evidence of progress and challenges in the Mexican health system. Health Policy Plann.

[25] Wagstaff A, Flores G, Hsu J, Smitz M-F, Chepynoga K, Buisman LR (2018). Progress on catastrophic health spending in 133 countries: a retrospective observational study. Lancet Global Heal.

[26] Wagstaff A, Doorslaer VE. Paying for health care: quantifying fairness, catastrophe, and impoverishment, with applications to Vietnam, 1993–98. World Bank Publications; 2001.

[27] Tracking universal health coverage: 2017 global monitoring report. World Health Organization and International Bank for Reconstruction and Development / The World Bank. 2017.

[28] Cylus J, Thomson S, Evetovits T (2018). Catastrophic health spending in Europe: equity and policy implications of different calculation methods. B World Health Organ.

[29] Knaul F, Arreola-Ornelas H, Wong R, Lugo-Palacios DG, Méndez-Carniado O (2018). Efecto del Seguro Popular de Salud sobre los gastos catastróficos y empobrecedores en México, 2004–2012. Salud Pública de México.

[30] Rosenbaum PR, Rubin DB (1983). Assessing Sensitivity to an Unobserved Binary Covariate in an Observational Study with Binary Outcome. J Royal Statistical Soc Ser B Methodol.

[31] Sosa-Rubí SG, Galárraga O, Harris JE (2009). Heterogeneous impact of the “Seguro Popular” program on the utilization of obstetrical services in Mexico, 2001–2006: A multinomial probit model with a discrete endogenous variable. J Health Econ.

[32] López-Manning M, García-Díaz R (2017). Doctors Adjacent to Private Pharmacies: The New Ambulatory Care Provider for Mexican Health Care Seekers. Value Health Reg Issues.

[33] Palmer N, Mueller DH, Gilson L, Mills A, Haines A (2004). Health financing to promote access in low income settings—how much do we know?. Lancet.

[34] Gomes C (2019). Health Systems in Latin America: Principal Components of Attention. Health.

[35] Reich MR (2020). Restructuring Health Reform. Mexican Style Heal Syst Reform.

[36] Kawabata K, Xu K, Carrin G (2002). Preventing impoverishment through protection against catastrophic health expenditure. Bull World Health Organ.

[37] Ekman B (2007). Catastrophic health payments and health insurance: Some counterintuitive evidence from one low-income country. Health Policy.

[38] Cavagnero E, Carrin G, Xu K, Aguilar-Rivera AM. Health Financing in Argentina: An Empirical Study of Health Care Expenditure and Utilization. Working Paper 8. Edited by: Financing IiH. Mexico: Instituto Nacional de Salud Pública; 2006.

[39] Wirtz VJ, Serván-Mori E, Heredia-Pi I, Dreser A, Ávila-Burgos L (2013). Factores asociados con la utilización y el gasto en medicamentos en México. Salud Pública Méx.

[40] Parker SW, Saenz J, Wong R (2018). Health Insurance and the Aging: Evidence From the Seguro Popular Program in Mexico. Demography.

[41] Navarro SM, Pelcastre-Villafuerte BE, Becerril-Montekio V, Serván-Mori E (2022). Overcoming the health systems’ segmentation to achieve universal health coverage in Mexico. Int J Heal Plan Manage.

[42] Michelson H, Muñiz M, DeRosa K (2013). Measuring Socio-economic Status in the Millennium Villages: The Role of Asset Index Choice. J Dev Stud.

