# Supplementary material for: Effective access to health care in Mexico
Source: BMC Health Serv Res. 2022 Aug 12;22:1027. doi: 10.1186/s12913-022-08417-0 (PMC9373534; doi:10.1186/s12913-022-08417-0)
Supplement: Supplementary file 3 — Additional file 3. [file 12913_2022_8417_MOESM3_ESM.docx]

**Figure A.1. Propensity Score Matching**

| Source: Author's calculations based on ENSANUT 2018. |
| --- |
