# Supplementary material for: Effective access to health care in Mexico
Source: BMC Health Serv Res. 2022 Aug 12;22:1027. doi: 10.1186/s12913-022-08417-0 (PMC9373534; doi:10.1186/s12913-022-08417-0)
Supplement: Supplementary file 2 [file 12913_2022_8417_MOESM1_ESM.docx]

Table A.1 Logit Estimates for Balancing Score

	Coefficient	z-statistic
Female	0.1614933 (0.00691)	23.37
Group age:		
Age 20 to 39 years	-0.1832305 (0.0092606)	-19.79
Age 40 to 59	-0.1612751 (0.0106085)	-15.2
Age 60 to 79	-0.3418459 (0.0125793)	-27.18
Married	-0.0339722 (0.0090256)	-3.76
Education:		
Primary education	0.3069046 (0.0093214)	32.92
Secondary education	0.2896045 (0.0096633)	29.97
High school education	-0.0136517 (0.0108272)	-1.26
Rural	0.5150567 (0.0082994)	62.06
Assets index	-0.244324 (0.0027752)	-88.04
Area (reference category south)		
North	-0.4257982 (0.0097824)	-43.53
Center	-0.1660193 (0.0080167)	-20.71
Constant	-0.2907278 (0.0097806)	-29.73
Number of observations	154, 838	
Pseudo R2	0.1516	
Log likelihodd test	33279.72	
Prob > chi2	0	

Source: Author's calculations based on ENSANUT 2018.

Notes: Standard error in parenthesis.
