# Supplementary material for: Effective access to health care in Mexico
Source: BMC Health Serv Res. 2022 Aug 12;22:1027. doi: 10.1186/s12913-022-08417-0 (PMC9373534; doi:10.1186/s12913-022-08417-0)
Supplement: Supplementary file 3 [file 12913_2022_8417_MOESM2_ESM.docx]

Table A.2 Logit Estimates for Balancing Score

	Mean			t-test	
	Treated	Control	%bias	t	p>t
Female	0.5326	0.52024	2.5	3.72	0
Group age:					
Age 20 to 39 years	0.28984	0.27807	2.6	3.92	0
Age 40 to 59	0.22084	0.22239	-0.4	-0.56	0.575
Age 60 to 79	0.10948	0.1101	-0.2	-0.3	0.765
Married	0.28142	0.26001	4.7	7.24	0
Education:					
Primary education	0.30828	0.31684	-1.9	-2.77	0.006
Secondary education	0.28806	0.27772	2.4	3.45	0.001
High school education	0.16016	0.16241	-0.6	-0.91	0.36
Rural	0.29332	0.30284	-2.2	-3.12	0.002
North	0.20155	0.19734	1	1.58	0.113
Center	0.43569	0.42452	2.3	3.38	0.001

Source: Author's calculations based on ENSANUT 2018.

Notes: Standard error in parenthesis.
